# Coagulation and Bleeding Management in Pediatric Extracorporeal Membrane Oxygenation: Clinical Scenarios and Review

**DOI:** 10.3389/fmed.2018.00361

**Published:** 2019-01-11

**Authors:** Lisa A. Hensch, Shiu-Ki Rocky Hui, Jun Teruya

**Affiliations:** ^1^Division of Transfusion Medicine & Coagulation, Texas Children's Hospital, Houston, TX, United States; ^2^Department of Pathology & Immunology, Baylor College of Medicine, Houston, TX, United States

**Keywords:** anti-Xa assay, acquired von Willebrand syndrome, antifibrinolytic agents, therapeutic plasma exchange, antithrombin, plasma hemoglobin, ECMO—extracorporeal membrane oxygenation, bivalirudin

## Abstract

Extracorporeal membrane oxygenation (ECMO) is a life-saving procedure that requires careful coagulation management. Indications for ECMO continue to expand, leading to more complicated patients treated by ECMO teams. At our pediatric institution, we utilize a Coagulation Team to guide anticoagulation, transfusion and hemostasis management in an effort to avoid the all-to-common complications of bleeding and thrombosis. This team formulates a coagulation plan in conjunction with a multidisciplinary ECMO team after careful review of all available laboratory data as well as the patient's clinical status. Here, we present our general strategies for ECMO management in various clinical scenarios and a review of the literature pertaining to coagulation management in the pediatric ECMO setting.

## Introduction

Over 7,000 ECMO cases were reported to the Extracorporeal Life Support Organization in 2016. Of these, nearly a third represent neonatal or pediatric patients (1). ECMO presents unique challenges to all teams involved in the management of these often very complicated patients. ECMO is most frequently employed in patients who are in imminent danger of dying from respiratory failure, cardiac failure or a combination of both. As both physician knowledge and comfort regarding this method of support increases, indications for ECMO continue to expand and relative contraindications seem to become less insurmountable. As a result, the coagulation obstacles faced by the teams managing ECMO patients have also become more varied.

Cannulation for ECMO may be planned, in which case underlying coagulation abnormalities can be largely corrected, or may occur during active extracorporeal cardiopulmonary resuscitation (ECPR). ECPR patients will often have larger coagulation deficits at the beginning of the ECMO run since these circuits may be primed with crystalloid rather than red blood cells (RBC) and fresh frozen plasma (FFP). In addition, there is little time to correct, or sometimes even assess, the degree of underlying hemostatic abnormalities for many of these patients. As the duration of support increases so do the risks of hemostatic and/or thrombotic complications. A variety of methods of coagulation support may be required including: alternative laboratory monitoring, changes in anticoagulation, transfusion support, utilization of various hemostatic agents, therapeutic pheresis and other therapies.

At our institution, ECMO patients are all treated by a multidisciplinary team composed of pediatric or cardiovascular surgeons, intensive care physicians, perfusionists, bedside nurses, pharmacists and transfusion medicine and coagulation pathologists. Other teams, such as pulmonology, cardiology, neonatology and infectious disease may be involved depending on the needs of each particular patient. We believe that this multidisciplinary approach has improved our ECMO service and provides the best care for these patients. ECMO has been utilized in ~50 patients per year over the last 3 years in our institution (range: 44–58). The majority of these are cardiovascular intensive care patients (57%) with an additional 23% of patients in the neonatal intensive care unit and 20% pediatric intensive care patients.

As we learn from each patient, we believe that it is important that we reflect on our experiences to evaluate what we can improve in future management strategies. Below we present a variety of clinical situations we have encountered as transfusion medicine and coagulation pathologists working with our ECMO teams.

## Coagulation Evaluation and Management

### Scenario 1

A non-coagulopathic pediatric patient with lung disease of uncertain etiology was placed on ECMO. Support continued for 72 days while the team completed workup and subsequent treatment. The course was complicated by multiple bleeding issues including: bleeding from the cannula site, oral oozing, profound epistaxis and gastrointestinal bleeding. Despite treatment with multiple hemostatic agents, heparin activity remained stable without major adjustments and full circuit change was never required. It was observed that antithrombin (AT) activity was increased at 150–170% for the majority of the ECMO course.

The above scenario, with the exception of persistently elevated antithrombin, is not uncommon in the setting of pediatric ECMO. At our institution, ECMO is frequently employed to support the respiratory function of patients while their underlying disease can be determined or treated. While cannulation in these circumstances does not present a major coagulation challenge, supporting these children for months on mechanical support becomes increasingly challenging as the risk for bleeding and thrombotic complications increases. For this reason, it is important to maintain consistent levels of anticoagulation and coagulation support.

### General Monitoring

Coagulation status is monitored using our ECMO panel (detailed in Table 1) which is obtained every 6 h. In addition, most patients are additionally monitored with daily rotational thromboelastogram (ROTEM) and plasma hemoglobin. The prothrombin time (PT) and partial thromboplastin time with hepzyme (PTT-hepzyme) tells the team about the underlying coagulation status. Based on these results, plasma may be used to correct underlying coagulopathy. These measurements also help the team determine if the patient is developing disseminated intravascular coagulation (DIC), a situation in which plasma exchange may be warranted. The PTT-hepzyme can also give the coagulation team important clues as to the existence of a hypercoagulable state. A shortened PTT-hepzyme may indicate an increased level of factor VIII (FVIII) and/or circulating thrombin, which increases the risk of thrombosis in both the patient and circuit. In contrast, an unexpectedly prolonged PTT-hepzyme, which is out of proportion to the PT, may additionally point to the presence of lupus anticoagulant. The timely identification of these hypercoagulable states pushes the team to increase heparin dosages or consider using plasma exchange to avoid increasing thrombotic complications.

**Table 1:** Institutional ECMO panel performed every 6 h.

**Test**	**Target**	**Reason**	**Reaction if outside of target**
**ECMO COAGULATION PANEL**			
Prothrombin Time (PT)	<17 s	Monitor underlying coagulopathy	Transfusion support with FFP
Partial Thromboplastin Time (PTT)	60–90 s	Target for heparin therapy	Increase/decrease heparin as indicated (if anti-Xa also out of target or is invalid)
PTT Hepzyme	Near normal range	Monitor underlying hypercoagulability, or coagulopathy	If above: consider FFP or testing for lupus anticoagulant If below: consider hypercoagulability due to increased factor VIII and/or circulating thrombin
Anti-Xa	<0.20–0.50 IU/mL	Institutional primary method for monitoring heparin therapy	Increase/decrease heparin as indicated
Antithrombin	>80–100%	Monitor need for antithrombin therapy if heparin effect not at target	Administer antithrombin concentrate (50 U/kg) if heparin effect below target
Platelets	>100,000/μL (100 × 10^9^/L)	Monitor bleeding risk	Transfusion support with platelets
Fibrinogen	>200 mg/dL (2 g/L)	Monitor bleeding risk	Transfusion support with cryoprecipitate
D-dimer	No target	Monitor clot formation/burden in circuit	Consider changing heparin target or circuit component change if rapidly increasing clinical assessment for acute thrombosis/bleeding

There is no consensus on the appropriate platelet count or fibrinogen level needed for ECMO patients. Indeed, frequent transfusion of platelets, while reducing the risk of bleeding complications, comes with a cost. Platelet factor 4 (PF4) binds to heparin, reducing its bioavailability and diminishing its effect. However, the foreign surfaces of ECMO also activate platelets, reducing their activities and lifespan. We recommend a platelet count of >100,000/μL (100 × 10^9^/L) for the majority of our patients on ECMO. This level can be adjusted based on bleeding or thrombotic complications. Our standard dose for patients on ECMO is 5–10 mL/kg for patients <35 kg or 1 apheresis platelet unit for patients >35 kg. We also tend to lower the threshold if the patient is not bleeding and/or this level cannot be reasonably maintained. Our fibrinogen target is >200 mg/dL (2 g/L). After cannulation, this level can generally be easily maintained without the need for frequent cryoprecipitate transfusion. When cryoprecipitate is used, the dose given is 1–2 units per 10 kg. Fibrinogen level may also be increased as a result of the acute phase reaction seen in the setting of inflammatory states or infection. It is important to note that an inability to maintain platelet and fibrinogen targets in patients who do not appear to have concurrent DIC or active bleeding is an important clue to rapid consumption occurring within the circuit and an indicator that circuit or component change should be considered.

### Monitoring Heparin Effect

Unfractionated heparin (heparin hereafter) is usually the anticoagulant of choice for ECMO, however, methods of anticoagulation monitoring in this setting vary widely among institutions (2). At our center, we monitor heparin effect by a variety of methods including: activated clotting time (ACT), anti-Xa assay, PTT, and ROTEM. Each of these is interpreted in light of the patient's clinical status, circuit status and other laboratory data to make the most informed decision possible with regards to the need for heparin adjustment. ACT is still used in a majority of centers in the United States and still remains available for point-of-care testing in our center. Benefits of using ACT include a rapid turnaround time and small sample size (3). It is important to remember, however, that the ACT is a global measure of hemostasis. As such, ACT results reflect not only heparin levels, but may also reflect coagulation activation and patient factors, such as hypothermia (4). Deficiency of platelets, platelet dysfunction, low fibrinogen, clotting factor abnormalities and hemodilution additionally affect the ACT (5). For the patient on ECMO, ACT results should be interpreted with these additional variables in mind. Platelet dysfunction or hypofibrinogenemia detected by the ACT may lead to under-heparinization and increased risk for thrombotic complications. Several studies have also found a lack of correlation between the ACT and heparin dosing (6, 7).

While we do utilize a variety of tests to assess heparin effect, we primarily rely on the anti-Xa assay for titration (8). The anti-Xa assay measures only the effects of heparin on factor Xa, and therefore is generally not affected by other coagulation abnormalities. In a pediatric study, Liveris et al. reported moderate to strong correlation between heparin dose and anti-Xa activity (6). O'Meara and colleagues described a monitoring protocol using anti-Xa every 6 h. This was found to be associated with fewer adjustments to heparin rates and decreased sampling requirements (7). This is particularly important in our neonatal patients who sometimes require frequent transfusions due to sampling requirements. Measurement of heparin effect using anti-Xa has also been shown to correlate better with circuit outcomes than the ACT (9). Finally, use of anti-Xa assay in conjunction with thromboelastography and AT was shown to reduce the need for transfusion and reduce hemorrhagic complications (10). However, like the ACT, the anti-Xa assay does have limitations. This is a chromogenic assay that relies on the detection of a colored compound at 405 nm (11). Higher levels of exogenously added bovine factor Xa remaining in the sample increase the color detected and reflect less heparin activity. Therefore, patient-related issues leading to samples with high plasma hemoglobin, high bilirubin and/or lipemia, will all reflect lower heparin levels than are actually present. Anti-Xa levels have been shown to be decreased by 0.03–0.05 IU/mL for every 100 mg/dL (1 g/L) increase in plasma hemoglobin or bilirubin increase by 6 mg/dL (103 μmol/L) (12). Again, it is important that clinicians managing ECMO patients are aware of these interferences so that heparin is not increased to supratherapeutic levels. ECMO clinicians using anti-Xa assays should also be aware if their institutional assay uses an exogenous source of AT. Particularly in neonatal and pediatric patients, assays with an exogenous source of AT are less likely to accurately reflect *in vivo* heparin status, as these patients will frequently have low levels of endogenous AT. In situations where anti-Xa levels are believed to be inaccurate, other methods for evaluating heparin effect should be considered.

The PTT is the preferred “back-up” method used by our group for monitoring heparin activity. Of note, the PTT may be physiologically prolonged in neonates, making established reference ranges unreliable. However, our clinical laboratory utilizes hepzyme, allowing us to look at the underlying status of the PTT, therefore, the desired target can be appropriately adjusted. PTT has been shown to correlate better with heparin dose and was associated with less bleeding when used to monitor pediatric patients as compared to ACT in one study (13). As mentioned above, the PTT may be affected by increased levels of FVIII which is a common finding among ECMO patients. A below target PTT, even with an appropriate anti-Xa assay, may indicate the need to increase the target anti-Xa level. In situations where there is disagreement between monitoring methods, the final determination regarding changes to anticoagulation is made only after a thorough clinical assessment and discussion about the status of both the patient and the circuit.

### Antithrombin (AT)

Similar to other coagulation issues in pediatric ECMO, the need for AT in this setting is an unresolved issue. Even among centers that do monitor and replace AT, there is wide variation in reported targets (2). Our institutional target for AT is >80–100% activity. When patients have lower than desired AT, we use a standard dose of 50 U/kg of Thrombate®. Thrombate® is human-derived AT that is approved for patients who have hereditary AT deficiency (14). However, neonatal patients have a physiologic deficiency of AT with normal levels as low as 39% of adult levels in the normal, term newborn (15). This physiologic decrease is further exacerbated in neonates with sepsis (16). Several studies have examined the effects of AT administration for neonatal and pediatric patients with varying results (17–22). In 121 pediatric patients, approximately a quarter of which were on ECMO, Ryerson et al. demonstrated higher anti-Xa assay levels, lower heparin requirements and reported no bleeding complications when AT was administered (19). When evaluating the administration of one vial of AT (1,000 U; averaging 241 U/kg) Ryerson et al. also reported no adverse events, though it is important to note that heparin dose was decreased by 50% at the time of administration (18). Other studies have confirmed lack of bleeding complications associated with AT treatment (20). Tighter control of anticoagulation with AT replacement is also reported; however, this did not affect the longevity of the ECMO circuit (17). In our own patients, we find that we can often avoid continuous increases in heparin dose and achieve more stable levels when using AT replacement. Nearly all patients in our institution treated with heparin receive at least one dose of AT concentrate. Our neonatal population largely requires at least once daily dosing, and not infrequently these patients receive two or more doses daily, especially at the beginning of the ECMO course. AT supplementation in our pediatric population varies widely, but in general, these patients require far less frequent dosing to maintain our targets. Curiously, we have identified a few ECMO patients who have had high-normal to elevated AT levels throughout the duration of ECMO without replacement. In our experience, these patients maintain stable heparin requirements with fewer needs for adjustment. The use of AT, an expensive pharmaceutical agent, has been shown to decrease the rate of heparin resistance in the setting of cardiac bypass (23), and may see increasing use in the setting of pediatric ECMO to avoid this complication, but further studies are needed. Also, as clinicians become more comfortable with the use of alternative anticoagulants, the question of whether or not to replace AT may become obsolete.

### Plasma Hemoglobin

Mechanical circulatory support with ECMO is associated with intravascular hemolysis. Hemolysis with measured plasma hemoglobin >50 mg/dL (0.5 g/L) is reported in ~10% of pediatric and neonatal patients and is seen in up to 14% of patients requiring ECPR in the neonatal population in the ELSO data (1). This is in stark contrast to reports from individual centers that suggest much higher rates of hemolysis in these populations (24, 25). Discrepancies in the reports of the number of patients with hemolysis is likely attributed to variability or lack of protocolized monitoring across institutions. Data also suggest that this problem is more commonly encountered in the pediatric population as hemolysis complicating ECMO is reported in only 5–6% of adult patients (1). Development of hemolysis is a serious complication in pediatric and neonatal ECMO for a variety of reasons. For example, high plasma free hemoglobin contributes to thrombosis by a variety of mechanisms. Increased scavenging of nitric oxide by free hemoglobin results in activation of endothelial cells and platelet activation/aggregation (26). The presence of free hemoglobin has also been shown to increase platelet adhesion by interaction with the A1 domain of von Willebrand factor (VWF), resulting in the formation of stable microthrombi in the setting of high shear stress (27).

The development of thrombi as a result of high plasma hemoglobin contributes to increased morbidity in ECMO patients. Risk factors associated with hemolysis include in-line hemofiltration, venous inlet pressure, type of oxygenator, and pump speed (24, 25, 28). Hemolysis may also occur secondary to patient factors and cannula size. Increased clotting within the circuit also contributes to hemolysis. In a recent study by Dalton et al., higher daily plasma hemoglobin levels were correlated with lower body weight, lower heparin dose (in IU/kg), and the development of renal insufficiency (28). Other studies have reported an increase in mortality associated with increased plasma hemoglobin levels (24, 25). Notably, the presence of high plasma hemoglobin interferes with monitoring of chromogenic anti-Xa assays, giving the false impression of low heparin levels, and putting the patient in risk of being over-anticoagulated as a result. For these reasons, we believe that monitoring plasma hemoglobin daily is critical to appropriate coagulation management in ECMO. At our institution, we use a cut-off of 150 mg/dL (1.5 g/L) using spectrophotometry (HemoCue Plasma/Low Hb Photometer, Brea, California) to start considering circuit change or plasma exchange to avoid the deleterious effects of ongoing hemolysis. It is also important that ECMO clinicians recognize the potential for preanalytical (hemolysis due to phlebotomy) and analytical (icteric or lipemic specimen) errors, and investigate accordingly if additional signs of hemolysis, such as hemoglobinuria or increasing LDH, are not present.

### Treatment of Bleeding

**Figure 1:**
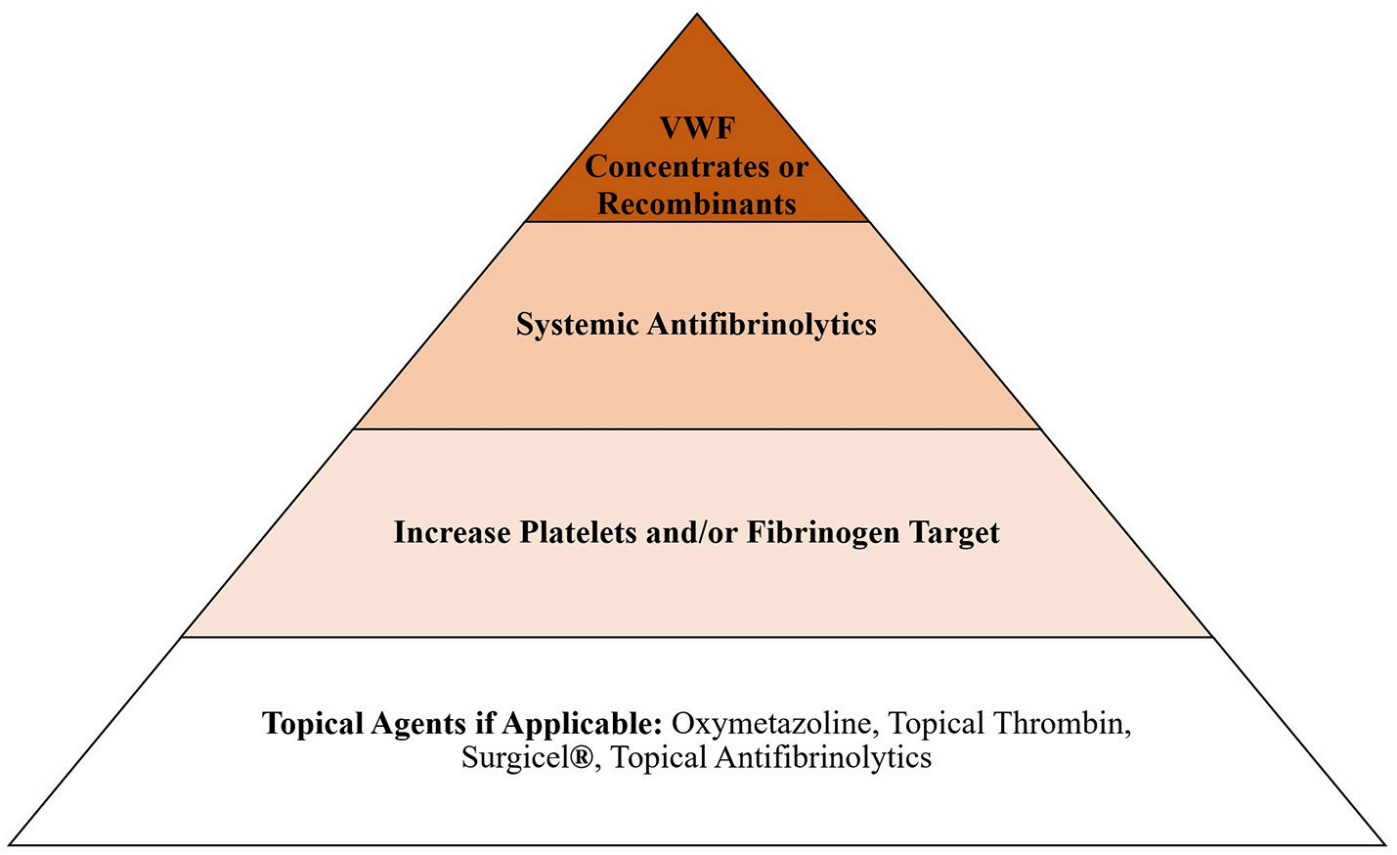
Escalation of agents used to treat bleeding in ECMO in our institution.

When assessing the balance between bleeding and clotting, our primary objective is always to treat bleeding, even if that includes the use of systemic hemostatic agents, such as tranexamic acid or von Willebrand factor, and change the circuit if treatment causes circuit failure. However, circuit change is not a benign process and can lead to coagulopathy due to hemodilution, volume overload and inflammatory changes associated with exposure to new foreign surfaces. These issues pose an additional threat to the patient and should be considered when choosing how to approach bleeding in the ECMO patient. For this reason, we try to use non-systemic agents whenever possible. Our general escalation in treatment for bleeding, after we consider reducing anticoagulation, is presented in Figure 1. Bleeding in ECMO occurs for a variety of reasons, including acquired von Willebrand syndrome (aVWS), thrombocytopenia, platelet dysfunction, hyperfibrinolysis and coagulopathy (8, 29).

#### Acquired von Willebrand Syndrome (aVWS)

AVWS is a well-known coagulation abnormality that developed among patients on ECMO support including pediatrics (30, 31). In our institution, as is described in literature (32), ECMO patients develop aVWS by day 1 post-ECMO which often progressively worsens over their ECMO course (33). AVWS in ECMO is thought to be related to loss of high molecular weight multimers (HMWM) due to high shear force within the circuit. However, despite its well-described association with ECMO, the clinical significance in regards to hemostasis remains controversial (34). From our experience, the best and most reliable method of determining presence and severity of aVWS is via multimer analysis as von Willebrand Factor (VWF) activity to antigen ratio does not always correlate well with degree of HMWM destruction. Our institutional standard is to perform weekly VWF testing which includes FVIII level, VWF antigen, VWF activity, VWF activity to antigen ratio and semi-quantitative multimer analysis. However, we do not empirically treat patients with aVWS, regardless of test results, unless there is clinically significant bleeding that cannot be attributed to more routine coagulopathy and/or the bleeding is unresponsive to standard treatment.

The main concern related to treating aVWS in absence of bleeding is the increased risk for thrombotic complications. Administration of exogenous VWF will inevitably increase both FVIII and VWF antigen and activity from baseline which can, in our experience, increase clotting in the ECMO circuit and the risk of thrombosis in the patient. The thrombotic risk is particularly high among patients already undergoing acute phase reaction with high baseline FVIII and VWF. For this reason, our facility performs weekly VWF analysis not only to determine severity of aVWS, but also to determine FVIII and VWF antigen levels and thereby better ascertain the thrombotic risk. This risk-to-benefit analysis is performed to determine if VWF supplementation is likely to be of benefit to the patient. VWF supplementation in the United States is available as HUMATE-P® (human derived VWF) and Vonvendi® (recombinant VWF). Vonvendi®, unlike HUMATE-P®, does not contain FVIII and therefore the risk for acute thrombosis from Vonvendi® infusion is theoretically lower. Nonetheless, Vonvendi® will still increase VWF antigen ultimately resulting in increase in FVIII over time. In our institution administering VWF supplementation is generally limited to two doses. The usual dosage for VWF infusion at our institution is 30 IU/kg for HUMATE-P® or 50 IU/kg for Vonvendi® which may be repeated once, 6 h after initial dose. Careful monitoring of the patient for new thrombosis and the ECMO circuit for new clot formation is strongly recommended. Fortunately, aVWS rapidly resolves after decannulation from ECMO (35).

#### Topical and Systemic Antifibrinolytics

Antifibrinolytics, ε-aminocaproic acid (Amicar®) and tranexamic acid, are frequently used in our institution in the setting of ECMO. We have had good success treating oral oozing with topical aminocaproic acid mouth swabs. Oxymetazoline (Afrin®) is typically used first for epistaxis, but for some cases of intractable bleeding, packing with gauze soaked in tranexamic acid has also been effective in our experience. We use systemic antifibrinolytics following some surgical procedures, such as congenital diaphragmatic hernia repair or tracheostomy, for 24–28 h. Amicar® has previously been shown to reduce surgical site bleeding (36). For Amicar®, our institution uses a bolus of 100 mg/kg followed by a continuous infusion of 30 mg/kg/hr in the perioperative setting. For tranexamic acid, a 4 mg/kg bolus followed by 1 mg/kg/hr is used.

## Alternative Treatments for Bleeding

### Scenario 2

A pediatric patient placed on ECMO for respiratory failure develops bleeding in the endotracheal tube and chest X-ray findings concerning for pulmonary hemorrhage. The patient is hemodynamically unstable and the team would like to avoid a circuit change at this time.

### Nebulized or Intrapulmonary Hemostatic Agents

Pulmonary hemorrhage is a bleeding complication seen in ECMO patients (1, 37). Nebulized tranexamic acid has been described as a treatment option for patients with intractable pulmonary hemorrhage (38–40). In our pediatric patients who develop pulmonary hemorrhage on ECMO, we recommend nebulized tranexamic acid. We use a dose of 250 mg (250 mg in 2.5 mL saline) for pediatric patients <25 kg, and 500 mg (500 mg in 5 mL saline) in patients over 25 kg administered three to four times daily, as has been previously described (40). This intervention often helps us to reduce or stop pulmonary hemorrhage without having to completely stop anticoagulation or use systemic hemostatic agents. Direct application of tranexamic acid using a bronchoscope (500 mg in 5 mL saline) has also been described with reported good response (39). In a prospective trial, Bafaqih and colleagues indicate that nebulized tranexamic acid works in about 50% of patients. In patients who had ongoing hemorrhage, therapy was escalated with the addition of recombinant activated factor VII (rFVIIa) at a dose of 35 μg/kg in 2 mL of saline, which achieved a response in an additional 33.3% (40). The rationale for the use of intrapulmonary rFVIIa is that intravenously administered rVIIa does not reach the alveoli, whereas intrapulmonary rFVIIa can directly interact with tissue factor in the alveoli to stop bleeding without impairing oxygen transport (41). Use of rFVIIa, either directly applied with bronchoscopy or by nebulizer, has been described by several groups for the treatment of pulmonary hemorrhage using a variety of doses (42–46). Currently, our practice is to use rFVIIa at a dose of 50 μg/kg in 50 mL of saline to be administered by bronchoscope in patients who have existing pulmonary hemorrhage or develop hemorrhage during bronchoscopy. We have not experienced adverse outcomes using tranexamic acid or rFVIIa in this manner, but further study on intrapulmonary hemostatic agents in the setting of ECMO should be performed.

## Therapeutic Plasma Exchange and Purpura Fulminans on ECMO

### Scenario 3

A pediatric patient presents with DIC and respiratory failure. The patient is cannulated for venovenous (VV) ECMO, but rapidly becomes unstable and requires conversion to venoarterial ECMO. Coagulopathy is aggressively treated with plasma, platelet and cryoprecipitate transfusion, however, coagulation targets cannot be maintained. Platelets remain <50,000/μL (50 × 10^9^/L) despite frequent transfusion. The patient also developed diffuse purpura on the arms and legs.

### Therapeutic Plasma Exchange on ECMO

There are increasing reports of therapeutic plasma exchange (TPE) with concurrent ECMO described in the literature. A retrospective analysis of 293 procedures performed in 76 patients, 53 pediatric, demonstrated that concurrent ECMO and TPE was technically possible and tolerable, with hypocalcemia being the most frequent complication (47). TPE is performed in conjunction with ECMO for category 1 or 2 indications as classified by the American Society for Apheresis (ASFA) guidelines. TPE with ECMO is most commonly described in patients with rejection of a transplanted organ, sepsis with multiple organ failure, or thrombocytopenia-associated multiple organ failure (TAMOF) (48–52). Sepsis with multiple organ failure and TAMOF are currently category III indications for TPE (53). TAMOF is characterized by organ failure, platelet count <50,000/μL (50 × 10^9^/L) and thrombotic microangiopathy. This is due to low levels of a disintegrin and metalloproteinase with thrombospondin motifs-13 (ADAMTS-13), increased ultra-large von Willebrand multimers, and resulting VWF-rich thrombi in the microvasculature (54). Patients with TAMOF are at particularly high risk of dying; however, TPE can replace ADAMTS-13 and reverse organ dysfunction (55). Other studies have demonstrated improved outcomes of TAMOF treated with TPE, particularly when performed early in the course of organ failure (56).

TPE has been performed for pediatric ECMO patients in our institution for a variety of reasons, including TAMOF. In the last year, nearly 15% of our ECMO patients were treated with concurrent plasma exchange. Table 2 lists the “ECMO-related” reasons that TPE would be considered in our institution. We frequently consider the use of TPE in patients with various coagulopathies. Patients who have extremely elevated fibrinogen levels (>700 mg/dL or 7 g/L) and elevated FVIII due to acute phase reaction are considered for plasma exchange to limit the detrimental effects of this hypercoagulable state to both patient and circuit. We have also performed pheresis for multiple patients with concurrent significant bleeding and clotting in order to “reset” their underlying coagulable state. TPE for both coagulation reset and hypercoagulable states are generally performed only once as a temporizing measure while other therapies are initiated. TPE for high plasma hemoglobin is performed as a last resort if hemolysis is not corrected after circuit or circuit component change (Figure S1). This has generally occurred as a result of known or suspected thrombosis in the cannula, or in neonatal patients with small cannulas that cannot be upsized. Hemoglobin levels of >13 g/dL (130 g/L) have also been associated with hemolysis (57). All TPE performed on ECMO at our institution uses 100% FFP for replacement. TPE can be performed using only heparin, in which case serial monitoring with ACTs is performed during the procedure and heparin is adjusted accordingly. For patients who are subtherapeutic or those who appear to be hypercoagulable, regional citrate anticoagulation (ACD) is used in addition to systemic heparin. Most patients are also placed on a continuous calcium drip during the procedure to avoid the complications of hypocalcemia (58). This may be considered due to the ACD in FFP or if ACD is used as adjunct anticoagulation. Successful TPE with ECMO has also been reported using bivalirudin as an anticoagulant (59).

**Table 2:** Institutional criteria for TPE on ECMO.

**Indications for therapeutic plasma exchange on ECMO**
Thrombocytopenia-associated multiple organ failure
Plasma hemoglobin >150 mg/dL (1.5 g/L)
Elevated bilirubin level interfering anti-Xa measurement
Elevated unconjugated bilirubin level leading to risk for kernicterus in the neonate
Presence of lupus anticoagulant
Markedly elevated fibrinogen and/or factor VIII
Hemostasis reset: concurrent uncontrolled bleeding and clotting

### Protein C Concentrate

Purpura fulminans can be seen in the setting of DIC and is characterized by the development of multiple purpura and ecchymosis due to thrombosis of small vessels. Consumption of clotting factors due to DIC leads to low levels of both anticoagulant and procoagulant factors. In particular, protein C (PC) levels in pediatric patients presenting with shock, DIC and infectious purpura are ~20% of adult levels (60). In patients who present to our institution and develop purpura fulminans, we advocate for replacement of PC. Ceprotin® is a PC concentrate that is approved for treatment of congenital PC deficiency in pediatric and adult patients (61). There have been multiple reports of off-label use in the setting of pupura fulminans (62–65). We use a dose of 50 U/kg three to four times daily, depending on baseline value and if the patient is receiving concurrent TPE. Other groups have described using continuous infusion and intermittent boluses (100 U/kg) (64, 65). If TPE is planned, administration of PC occurs 6–8 h before and after TPE at our institution. In our opinion, this has limited the spread and duration of purpura and resulted in less tissue necrosis in these patients. A reduction in predicted morbidity and mortality in patients with meningococcemia and purpura fulminans when receiving PC was reported by White et al. (63) Similarly, another study reported improvement in laboratory markers of DIC and subjective improvement in patients when PC was administered to patients with infectious purpura fulminans caused by a variety of bacterial species (62). A retrospective study of patients receiving PC concentrate in Germany performed by Veldman et al. reported that there were fewer than expected dermatoplasties and amputations, and that PC administration was well-tolerated (65). These data suggest that PC should be considered as part of the management plan in patients who present with purpura fulminans. Of four patients recently treated with concurrent TPE and PC concentrate at our institution, all survived the acute episode of TAMOF with purpura fulminans, and 75% ultimately survived to decannulation and discharge.

## Alternative Anticoagulation

### Scenario 4

A pediatric patient presented with congestion, fever and shortness of breath. He was placed on mechanical ventilation, and soon thereafter cannulated for VV ECMO. A complete circuit change had to be performed within the first 12 h. Despite anti-Xa levels between 0.4 and 0.6 IU/mL, and PTT averaging >90 s, there was rapid reaccumulation of clot within the second circuit over the next 12 h. Prior to second circuit change, anticoagulation was switched to bivalirudin with PTT target of 60–80 s. No further circuit changes were required over the next 7 days of ECMO.

### Heparin

Heparin has traditionally been the anticoagulant of choice for ECMO, with the only big exception being patients with known or suspected heparin-induced thrombocytopenia (HIT). The primary mechanism of action for this anticoagulant by augmenting the effects of AT on factors Xa and IIa. Despite variability in monitoring and individual patient responses to heparin, heparin remains popular because of its rapid onset of action, average 90 min half-life and availability of a reversal agent (protamine) (8). As mentioned above, a number of factors affect the efficacy of heparin. Binding to proteins, such as PF4 and collagens, nonspecific binding to platelets, and accelerated clearance of AT due to ECMO can all contribute to heparin resistance (66). At our institution, we define heparin resistance as rapidly increasing clot burden despite adequate anticoagulation or inability to achieve therapeutic anticoagulation despite heparin dose of >40–50 IU/kg/hr. Heparin resistance can lead to rapid circuit failure. Figure S2 depicts an oxygenator that rapidly failed due to clotting, along with other common sites of fibrin deposition within the ECMO circuit.

### Bivalirudin

Bivalirudin is a direct thrombin inhibitor that is FDA approved for use in adults undergoing percutaneous intervention (67). However, primarily because of HIT, it has seen increasing use in the setting of ECMO. HIT is characterized by a rapid decrease in platelet count 5–14 days after exposure to heparin and can have life-threatening thrombotic complications. Of note, this happens more frequently in the adult population. In our pediatric institution, we have not had to change anticoagulant for ECMO due to HIT, but have switched due to heparin resistance. In 2018, bivalirudin was used as an anticoagulant for ~12% of our ECMO patients. Like heparin, bivalirudin has a quick onset of action and short half-life (8), making it ideal for use in the setting of ECMO. In addition, bivalirudin also has the advantage of acting on clot-bound thrombin, as opposed to heparin. However, there is a lack of physician comfort with this anticoagulant, which may be at least partially attributed to the fact that there is no reversal agent. Another concern related to the use of bivalirudin is the inability to continue monitoring underlying coagulopathy as the PT will also become prolonged in this setting. More frequent variation in the PTT using heparin as an anticoagulant vs. bivalirudin has been identified (68). In a separate, retrospective study, Ranucci et al. demonstrated that use of bivalirudin in ECMO was associated with less total blood loss and fewer transfusion needs (69). They additionally demonstrated that costs are reduced in bivalirudin patients, despite the higher cost of this anticoagulant as compared to heparin (69). A systematic review of the available publications for ECMO anticoagulation using bivalirudin determined that there is wide variability in how bivalirudin is used, particularly when it comes to bolus dosing, targets, and infusion rate (70). Bivalirudin therapy at our center is monitored using the PTT with a target of 60–90 s, which may be adjusted based on clot burden and individual patient characteristics. Therapy is initiated without bolus dose, typically at an infusion rate of 0.15–0.20 mg/kg/hr, which is similar to the published pediatric data (70, 71). We have not identified a change in the rate of bleeding complications when using bivalirudin as compared to heparin. In our experience, bivalirudin dose requirements are generally stable for short-term ECMO runs. However, increasing dose requirement over time has been demonstrated (71). Further study on the use of bivalirudin in this setting is warranted. The use of other direct thrombin inhibitors, argatroban and lepirudin, in the setting of pediatric ECMO has also been described (72, 73).

## Conclusion

A “one size fits all” approach to coagulation management in pediatric ECMO patients is not practical. Strategies that may work for one patient may prove detrimental in others. Each patient's status must be evaluated in light of their individual laboratory data and clinical characteristics. For this reason, our institution integrates a Coagulation Team into the multidisciplinary group managing ECMO patients. The Coagulation Team is an important component of the multidisciplinary team to incorporate laboratory findings with clinical findings. Our assessment includes frequent monitoring of heparin activity and underlying coagulation status. In addition, based on the clinical and laboratory importance of elevated free plasma hemoglobin, this is monitored daily. At all times, the team must be aware of factors that interfere with laboratory data. For appropriate management of ECMO patients, laboratory support is imperative and coagulation and hematology labs should be offered around-the-clock. Despite careful management, treatment with hemostatic agents in ECMO is often necessary and should be carried out in a thoughtful manner, considering both the consequences of ongoing bleeding and the potential harmful effects of circuit change. Alternative, non-systemic methods of managing bleeding should be considered when available. TPE is an emerging modality used to treat coagulopathy in ECMO patients, particularly in the setting of TAMOF. There is an increasing amount of evidence available for the use of bivalirudin in ECMO patients in the setting of HIT or heparin resistance, and this anticoagulant should be considered by clinical teams when heparin is contraindicated or its effects are inadequate. While we continue to learn more about ECMO each day, ongoing clinical observation, reporting, and research is needed to determine the best course of action when treating these critically ill pediatric patients.

## Author Contributions

LH was primarily responsible for the literature search, review of literature, and composition of the manuscript. SH and JT contributed to literature review, composition of the manuscript, and critical review of the manuscript. LH, SH, and JT all contributed to the institutional protocols described within the paper.

### Conflict of Interest Statement

The authors declare that the research was conducted in the absence of any commercial or financial relationships that could be construed as a potential conflict of interest.

## Supplementary Material

Figure S1 Plasma recovered from a patient with free plasma hemoglobin >150 mg/dL (1.5 g/L) by TPE.

Figure S2 Clotting within ECMO circuit. Clots during ECMO frequently occur in the oxygenator **(A)**, centrifugal pump **(B)**, lines **(C)**, and connection sites **(D)**.
