# Supplementary figures and images for: Coagulation and Bleeding Management in Pediatric Extracorporeal Membrane Oxygenation: Clinical Scenarios and Review
Source: Front Med (Lausanne). 2019 Jan 11;5:361. doi: 10.3389/fmed.2018.00361 (PMC6340094; doi:10.3389/fmed.2018.00361)

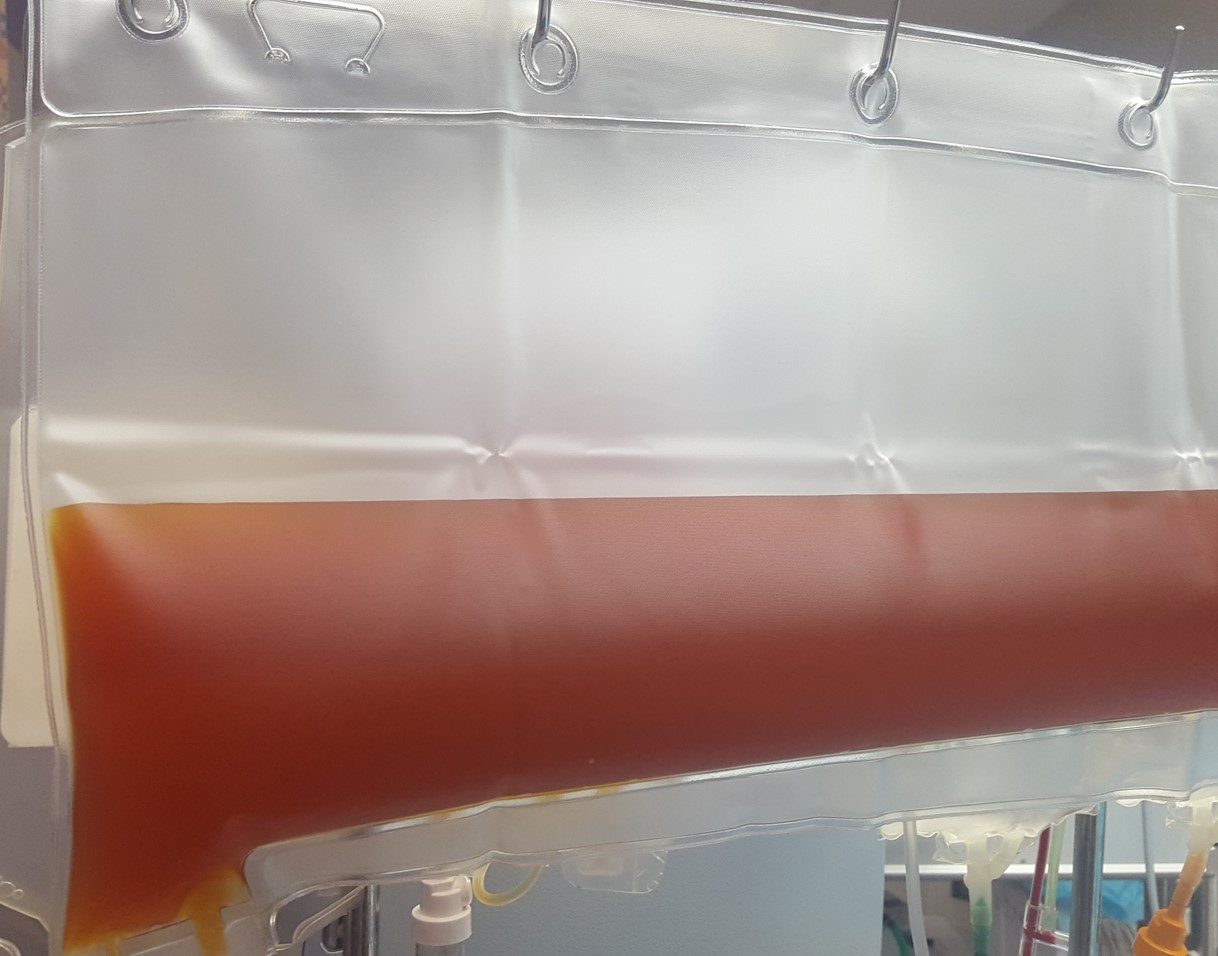

Supplement: Figure S1 — Plasma recovered from a patient with free plasma hemoglobin >150 mg/dL (1.5 g/L) by TPE. [file Image_1.jpg]

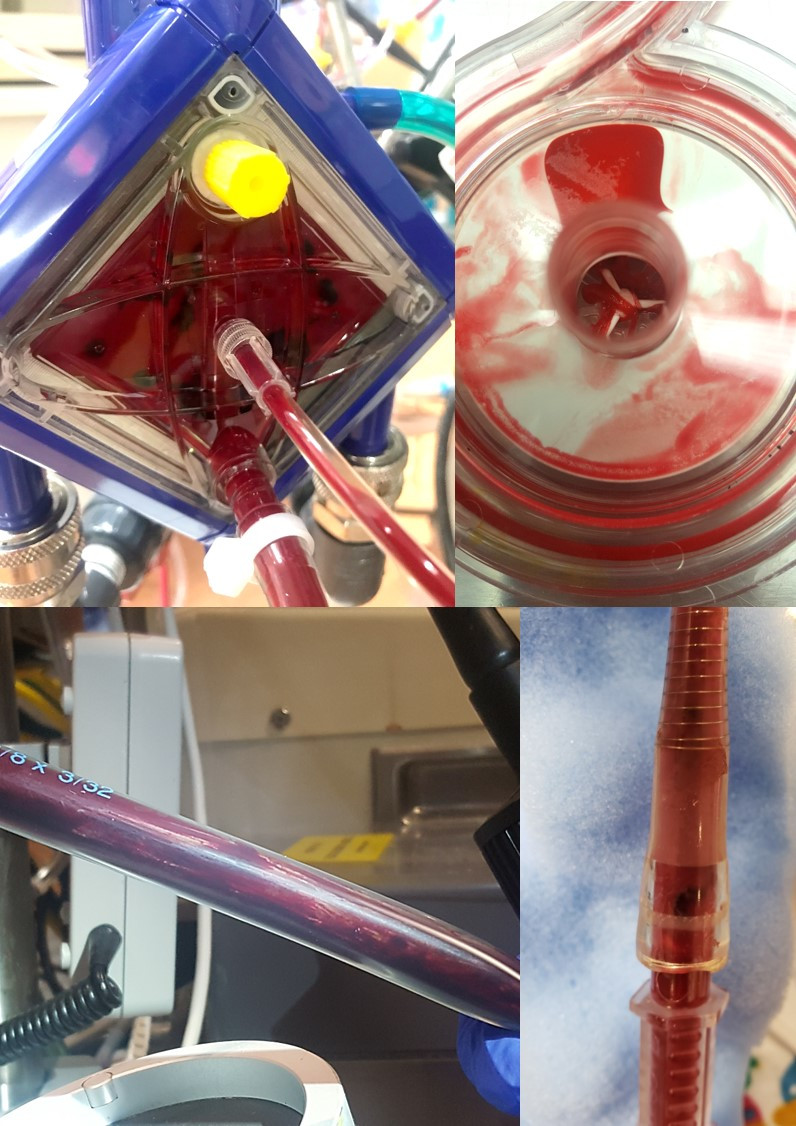

Supplement: Figure S2 — Clotting within ECMO circuit. Clots during ECMO frequently occur in the oxygenator (A), centrifugal pump (B), lines (C), and connection sites (D). [file Image_2.jpg]
